# Lixivaptan, a New Generation Diuretic, Counteracts Vasopressin-Induced Aquaporin-2 Trafficking and Function in Renal Collecting Duct Cells

**DOI:** 10.3390/ijms21010183

**Published:** 2019-12-26

**Authors:** Annarita Di Mise, Maria Venneri, Marianna Ranieri, Mariangela Centrone, Lorenzo Pellegrini, Grazia Tamma, Giovanna Valenti

**Affiliations:** 1 Department of Biosciences, Biotechnologies and Biopharmaceutics, University of Bari, 70125 Bari, Italy; 2 Palladio Biosciences, Inc., Newtown, PA 18940, USA

**Keywords:** V2R, vaptans, vasopressin, lixivaptan, Aquaporin-2

## Abstract

Vasopressin V2 receptor (V2R) antagonists (vaptans) are a new generation of diuretics. Compared with classical diuretics, vaptans promote the excretion of retained body water in disorders in which plasma vasopressin concentrations are inappropriately high for any given plasma osmolality. Under these conditions, an aquaretic drug would be preferable over a conventional diuretic. The clinical efficacy of vaptans is in principle due to impaired vasopressin-regulated water reabsorption via the water channel aquaporin-2 (AQP2). Here, the effect of lixivaptan—a novel selective V2R antagonist—on the vasopressin-cAMP/PKA signaling cascade was investigated in mouse renal collecting duct cells expressing AQP2 (MCD4) and the human V2R. Compared to tolvaptan—a selective V2R antagonist indicated for the treatment of clinically significant hypervolemic and euvolemic hyponatremia—lixivaptan has been predicted to be less likely to cause liver injury. In MCD4 cells, clinically relevant concentrations of lixivaptan (100 nM for 1 h) prevented dDAVP-induced increase of cytosolic cAMP levels and AQP2 phosphorylation at ser-256. Consistent with this finding, real-time fluorescence kinetic measurements demonstrated that lixivaptan prevented dDAVP-induced increase in osmotic water permeability. These data represent the first detailed demonstration of the central role of AQP2 blockade in the aquaretic effect of lixivaptan and suggest that lixivaptan has the potential to become a safe and effective therapy for the treatment of disorders characterized by high plasma vasopressin concentrations and water retention.

## 1. Introduction

Vaptans are non-peptide and orally active vasopressin receptor antagonists which act both on Vasopressin V1a Receptor (V1aR) and V2R or selectively on V2R. In the kidney, vasopressin binds V2R receptor and activates the cAMP signal transduction pathway, which in turn results in the transport of AQP2-containing vesicles to the apical plasma membrane and consequent water reabsorption [1,2,3]. Translocation of AQP2 is associated with cAMP-dependent kinase (PKA) activation, which is responsible for an increase in serine 256 (pS256) phosphorylation [4,5]. By blocking the V2 receptor, V2R antagonists cause aquaresis with a consequent decrease in urinary osmolality and a rapid correction of serum hyponatremia [6]. 

In 2009, tolvaptan—a specific V2R antagonist—was approved by the Food and Drug Administration (FDA) for the treatment of euvolemic and hypervolemic hyponatremia associated with heart failure, cirrhosis, and SIADH (syndrome of inappropriate antidiuretic hormone secretion) [7,8,9]. Moreover, tolvaptan is the first drug approved by regulatory authorities in Japan, Europe, Canada, and the USA for the treatment of ADPKD (autosomal dominant polycystic kidney disease) in patients at high risk of progression to ESRD (end stage renal disease). Tolvaptan was shown to delay the rate of decline in renal function in ADPKD patients, presumably by inhibiting intracellular cAMP production in the kidney and slowing the rate of renal volume increase [10,11]. Although the therapeutic benefit of tolvaptan has been widely reported, treatment with tolvaptan has also been associated with important adverse effects, including polyuria and potentially severe liver injury [10,11].

Recently, a novel vaptan, lixivaptan—an orally active, non-peptide, competitive, and selective antagonist for the vasopressin V2 receptor—completed phase 2 clinical studies in patients with ADPKD [12]. Lixivaptan is functionally equivalent to tolvaptan and has shown similar efficacy in patients with hyponatremia and on key pharmacodynamic biomarkers that are predictive of efficacy in ADPKD [12,13]. Recently, the efficacy of lixivaptan as a potential therapy for the treatment of ADPKD was investigated in PCK rats, a validated animal model of PKD. In this animal model, animals treated with lixivaptan showed decreased kidney weight, cysts and cAMP levels along with a reduction in plasma creatinine, indicating preserved renal function, compared to control animals [14]. Importantly, lixivaptan has shown no evidence of liver toxicity in the clinical studies conducted so far for the treatment of water retention disorders, specifically hyponatremia associated with CHF (congestive heart failure) and SIADH.

In the present study, we investigated in vitro the molecular mechanisms of the aquaretic effect of lixivaptan. To this end, renal collecting duct principal cells (MCD4) stably transfected with V2R and AQP2 were used. We provide the first demonstration of the central role of AQP2 blockade in the aquaretic effect of lixivaptan and suggest that lixivaptan has the potential to become a safe and effective therapy for the treatment of disorders characterized by high plasma vasopressin concentration and water retention.

## 2. Results 

### 2.1. Effect of Lixivaptan on cAMP Levels in MCD4 Cells

**Figure 1:**
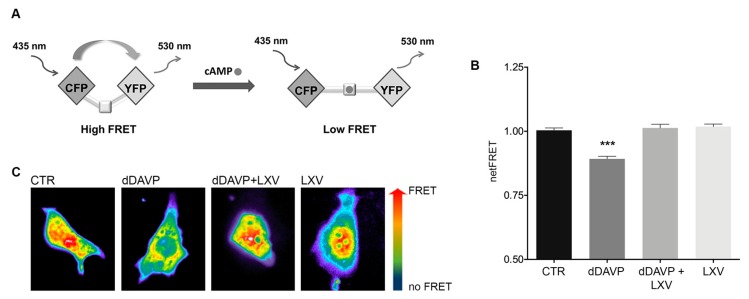
Measurements of intracellular cAMP by FRET. (**A**) Schematic model showing a FRET probe containing the cAMP-binding sequence of Epac1 sandwiched between ECFP (donor) and EYFP (acceptor). Binding of cAMP to the Epac1 results in an intermolecular steric conformation change causing an increase in the distance between the fluorescent donor and the acceptor, thereby decreasing the FRET process. (**B**) dDAVP stimulation significantly increased cAMP levels with respect to cells left untreated or in presence of lixivaptan (LXV), with or without dDAVP (*** *p* < 0.0001, *n* = 160 cells). Co-treatment with LXV prevented cAMP increase induced by dDAVP. All data were analyzed by one-way ANOVA followed by Newman–Keuls multiple comparisons test and are expressed as means ± SEM. (**C**) Representative transfected cells with H96 probe showing the FRET signal (ratio 535/30 nm) depicted in false color.

Since vasopressin binding to V2R is associated with an increase in intracellular cAMP levels, the effect of lixivaptan on cAMP levels was investigated with FRET-based EPAC biosensors, as previously described [15]. The FRET probe H96 contains the cAMP-binding sequence of Epac1 sandwiched between ECFP (donor) and EYFP (acceptor). Binding of cAMP to the Epac1 sequence causes an intermolecular steric conformation change, resulting in a relevant increase in the distance between the fluorescent donor and the acceptor, thereby decreasing the FRET process (Figure 1A). MCD4 cells stably transfected with human V2R and AQP2 [16] were exposed to dDAVP stimulation in the presence or in the absence of lixivaptan (100 nM). Based on preliminary experiments, 100 nM lixivaptan was chosen as the appropriate minimal concentration displaying a clear inhibitory effect on dDAVP-induced increase in cAMP levels in MCD4 cells (data not shown). dDAVP stimulation induced a clear increase in cAMP, depicted as a decrease in netFRET (CTR = 1 ± 0.009, *n* = 205 cells; dDAVP = 0.892 ± 0.009, *n* = 160 cells). Treatment with lixivaptan completely abolished the effect of dDAVP on cAMP (dDAVP+LXV = 1.013 ± 0.015, *n* = 250 cells) (Figure 1B). Lixivaptan alone did not cause any changes in intracellular cAMP levels (LXV = 1.018 ± 0.01, *n* = 150 cells).

### 2.2. Lixivaptan Prevents the Increase in pS256-AQP2 and AQP2 Translocation to the Plasma Membrane in Response to dDAVP

**Figure 2:**
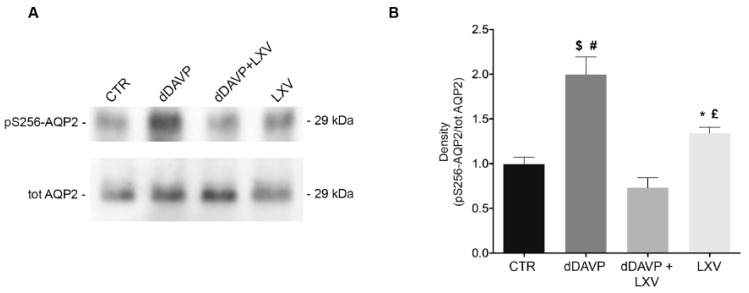
Effect of lixivaptan on pS256-AQP2 levels. (**A**) Equal amount of proteins from MCD4 cells were immunoblotted for evaluation of pS256-AQP2 and total AQP2 levels. (**B**) Statistical analysis revealed that lixivaptan prevented the increase of AQP2 phosphorylated at S256 induced by dDAVP. No alterations in pS256-AQP2 levels were observed by the sole treatment with lixivaptan. dDAVP alone induced a significant increase in pS256-AQP2 compared to cells under basal condition. Data are expressed as means ± SEM and were analyzed by one-way ANOVA followed by Newman–Keuls multiple comparisons test ($ *p* < 0.0001 dDAVP vs. dDAVP+LXV, # *p* < 0.001 dDAVP vs. CTR, £ *p* < 0.01 LXV vs. dDAVP, * *p* < 0.05 LXV vs. dDAVP+LXV, n.s. CTR vs. dDAVP+LXV or LXV; *n* = 5).

Phosphorylation of AQP2 at ser256 is a key signaling event for the relocation of AQP2 to the plasma membrane. On this basis, we next evaluated the effect of lixivaptan on pS256-AQP2. As expected, dDAVP significantly increased pS256-AQP2 compared to control (CTR = 1.000 ± 0.078, *n* = 5; dDAVP = 1.996 ± 0.2, *n* = 5). Co-treatment with lixivaptan prevented the increase in pS256-AQP2 levels, consistent with its action as V2R antagonist (dDAVP+LXV = 0.732 ± 0.11, *n* = 5). Treatment with lixivaptan alone did not alter basal pS256-AQP2 levels (LXV = 1.342 ± 0.064, *n* = 5) (Figure 2B). 

### 2.3. Lixivaptan Abolishes the Increase in Osmotic Water Permeability in Response to dDAVP

**Figure 3:**
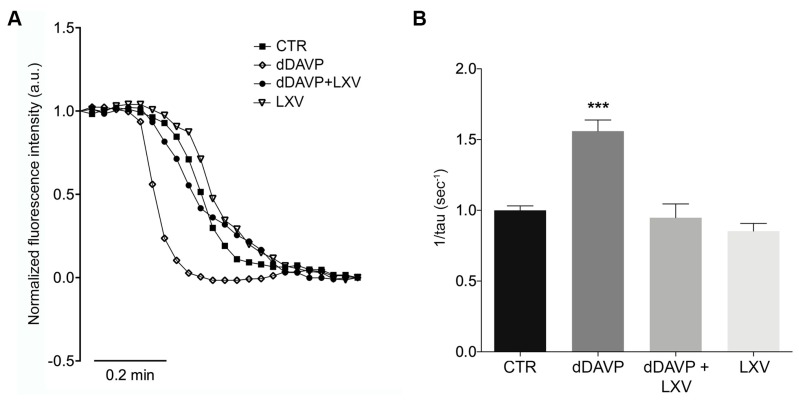
Effect of lixivaptan on osmotic water permeability. (**A**) Representative normalized traces of the time course of alterations in the osmotic water permeability in response to exposure to a hyperosmotic solution, followed as modulation of normalized calcein fluorescence intensity. Osmotic permeability measurements studies in MCD4 cells expressing human V2R and AQP2 were performed as described in Materials and Methods. (**B**) The temporal osmotic response is indicated as 1/τ (s^−1^). Lixivaptan prevented dDAVP-induced cell swelling (*** *p* < 0.0001). Data were analyzed by one-way ANOVA followed by Newman–Keuls multiple comparisons test and are expressed as means ± SEM.

AQP2 phosphorylation at ser256 is considered a crucial post-translational modification for the insertion of AQP2 into the plasma membrane, which in turn results in an increase in osmotic water permeability. Video imaging experiments were next performed to test the effect of lixivaptan on the time course of osmotic water permeability in response to dDAVP. Cells were grown on Ø40-mm glass coverslips and loaded with membrane-permeable calcein-AM. Fluorescence measurements were taken in the presence of either iso- or hyperosmotic solutions. dDAVP treatment induced a significantly higher temporal osmotic response (reported as 1/s) compared to untreated cells (CTR = 1.000 ± 0.031, *n* = 120 cells; dDAVP = 1.56 ± 0.078, *n* = 180 cells) (Figure 3B). Conversely, as expected for a V2R antagonist, lixivaptan prevented the increase in osmotic water permeability in response to dDAVP, indicating that lixivaptan inhibits AQP2 translocation and insertion into the plasma membrane (dDAVP+LXV = 0.948 ± 0.098, *n* = 120 cells). Lixivaptan alone did not show any effect on the basal osmotic water permeability (LXV = 0.853 ± 0.054, *n* = 130 cells).

## 3. Discussion

In this study, we report the first in vitro demonstration of the central role of AQP2 blockade in the aquaretic effect of lixivaptan. We provide evidence that lixivaptan prevents AQP2 trafficking to the apical membrane and phosphorylation at ser-256, as well as the increase in osmotic water transport in response to vasopressin in mammalian collecting duct principal cells.

Lixivaptan had been in development for the treatment of water retention disorders, specifically hyponatremia associated with CHF and SIADH. It was studied in 36 clinical studies and was administered to more than 1600 subjects as part of this clinical development program. Lixivaptan safely and effectively corrected serum sodium concentrations in patients with euvolemic hyponatremia [13,17]. 

To date, only tolvaptan has been proven to delay renal function decline in two phase 3 clinical studies in ADPKD [10,11] and has been approved for the treatment of ADPKD in Japan, Europe, Canada and the USA. In USA, the FDA has restricted the use of tolvaptan to a stringent risk management program because of tolvaptan’s potential to cause serious and potentially life-threatening idiosyncratic hepatotoxicity [14]. Quantitative system toxicology modeling simulations provided a prediction of liver effects elicited by the two compounds [18,19]. Specifically, lixivaptan showed a superior safety profile compared to tolvaptan with respect to the potential to cause liver injury.

Although the increased serum sodium concentrations elicited by lixivaptan administration in patients with euvolemic hyponatremia [17] and in cirrhosis patients with ascites [20] is likely a consequence of an aquaretic effect of lixivaptan, to date no direct in vitro evidence has been provided about the effect of lixivaptan on the vasopressin-induced AQP2 trafficking in collecting duct principal cells. 

We demonstrate here that lixivaptan prevents dDAVP-induced increased cAMP levels (Figure 1) and ser256-AQP2 phosphorylation in MCD4 cells stably expressing V2R and AQP2 (Figure 2). Real-time fluorescence kinetic measurements demonstrated that this effect is associated with the prevention of dDAVP-induced increase in osmotic water permeability (Figure 3). Using similar technical approaches, we have previously showed in MDCK cells expressing endogenous V2R, that tolvaptan prevented dDAVP-induced increase in ser256-AQP2 and osmotic water permeability [21]. 

Based on these results, the impairment of the V2R-PKA-AQP2 axis elicited by lixivaptan treatment appears to play a pivotal role in the aquaretic effect of lixivaptan and in general for vaptans acting as V2R antagonists. These data, together with the evidence of serum sodium levels increase in patients with euvolemic or hypervolemic hyponatremia upon treatment with lixivaptan, suggest that lixivaptan could be a promising effective therapeutic treatment for water retention disorders.

## 4. Materials and Methods

### 4.1. Chemicals and Reagents

Desmopressin (dDAVP) was purchased from Sigma (Sigma-Aldrich, Milan, Italy). Calcein-AM was bought from Molecular Probes (Life Technologies, Monza, Italy). Lixivaptan was kindly gifted from Palladio Biosciences Inc. (Palladio Biosciences Inc., Newtown, PA, USA). 

Cell culture media and FBS (fetal bovine serum) were from GIBCO (Life Technologies, Monza, Italy). Antibiotics were from Calbiochem (Merck Millipore, Milan, Italy). Super Signal^®^ West Pico Chemiluminescent Substrate was from Thermo Scientific (Thermo Fisher Scientific, Waltham, MA, USA) and used for the ChemiDoc System (Bio-Rad Laboratories, Milan, Italy). 

### 4.2. Antibodies

Total AQP2 antibodies (Pre-C-tail Ab), against the 20-amino acid residue segment situated just N-terminal from the poly-phosphorylated region of rat AQP2 (CLKGLEPDTDWEEREVRRRQ), and pS256-AQP2 antibodies were kindly gifted by Peter Deen. Secondary goat anti-rabbit horseradish peroxidase-coupled antibodies were obtained from Santa Cruz Biotechnologies (Tebu-Bio, Milan, Italy). Secondary goat anti-rabbit Alexa488 conjugated antibodies were from Molecular Probes (Thermo Fisher Scientific, Waltham, MA, USA).

### 4.3. Cell Culture and Treatments

Mouse Cortical Collecting Duct cells (MCD4), stably expressing human AQP2 and V2R, generated as described elsewhere [16,22], were grown in Dulbecco’s modified Eagle’s medium (DMEM/F12) supplemented with 5% (*v*/*v*) fetal bovine serum, 1% (*v*/*v*) L-glutamine, 1% penicillin/streptomycin, 5 µM dexamethasone, 400 µg/mL G418 (for AQP2 resistance) and 1 µg/mL puromycin (for V2R resistance) at 37 °C in 5% CO_2_.

After overnight treatment with indomethacin (5 × 10^−5^ M), cells were left under basal condition or stimulated with 100 nM dDAVP for 1h and/or treated with 100 nM lixivaptan for 1h. Addition of the prostaglandin synthesis inhibitor indomethacin was needed to reduce basal cAMP and AQP2-pS256 levels and was present in all treatments.

### 4.4. Cell Preparations

MCD4 cells were seeded onto 60-mm dishes and were left under basal condition or stimulated with 100 nM dDAVP for 1 h and/or treated with 100 nM lixivaptan for 1h. Subsequently, cells were homogenized in cell fractionation buffer (20 mM NaCl, 130 mM KCl, 1 mM MgCl_2_, 10 mM HEPES, pH 7.5) in the presence of proteases (1 mM PMSF, 2 mg/mL leupeptin and 2 mg/mL pepstatin A) and phosphatases (10 mM NaF and 1 mM sodium orthovanadate) inhibitors. The resulting homogenates were sonicated at 80% amplitude for 10 s. Cellular debris was removed by centrifugation at 12,000× *g* for 10 min at 4 °C. The supernatants were collected and used for immunoblotting experiments.

### 4.5. Fluorescence Resonance Energy Transfer Measurements

To evaluate intracellular cAMP levels, fluorescence resonance energy transfer (FRET) experiments were performed. Briefly, MCD4 cells were seeded onto 20-mm glass coverslips at 37 °C, 5% CO_2_ and transiently transfected with a plasmid encoding the H96 probe containing the cAMP binding sequence of Epac1 between cyan fluorescent protein (CFP) and cp173Venus-Venus (gift from Dr. K. Jalink). Experiments were performed 48 h after transfection. Cells were left under basal condition or stimulated with 100 nM dDAVP for 1 h and/or treated with 100 nM lixivaptan for 1 h. 

Visualization of ECFP- and/or EYFP-expressing cells and detection of FRET was performed on an inverted microscope (Nikon Eclipse TE2000-S), equipped with a monochromator controlled by Metamorph^®^ Microscopy Automation and Image Analysis Software (Molecular Devices, LLC, San Jose, CA, USA). ECFP was excited at 436 nm and EYFP at 500 nm. All images were aligned and corrected for background in the emission windows for FRET (535/30 nm), ECFP (475/30 nm), and EYFP (535/26 nm). Each image was further corrected for ECFP crosstalk and EYFP cross-excitation as shown by Rodighiero [23,24]. Thus, netFRET = IFRETbg − ICFPbg × k_1_ − IYFPbg (K_2_ − αK_1_)]/(1 − δK_1_), where IFRETbg, ICFPbg, and IYFPbg are the background-corrected pixel grey values measured in the FRET, ECFP, and EYFP windows, respectively; K_1_, K_2_, α, and δ are calculated to evaluate the crosstalk between donor and acceptor. Specifically, α = M_Dy_/M_DAy_, K_2_ = M_IAy_/M_DAy_, δ = M_Dy_/M_IAy_, determined in cells expressing only YFP (y), and K_1_ = M_IAc_/M_Dc_, determined in cells expressing only CFP (c). M indicates the measured fluorescence, corrected for background, M_D_ represents donor excitation with donor emission, M_IA_ donor excitation with acceptor emission, and M_DA_ acceptor excitation with acceptor emission, as described by van Rheenen [25].

### 4.6. Gel Electrophoresis and Immunoblotting 

Proteins were separated on 12% Bis-Tris acrylamide gels under reducing conditions. Protein bands were electrophoretically transferred onto Immobilon-P membranes (Merck Millipore, Milan, Italy) for western blot analysis, blocked in TBS–Tween-20 containing 3% bovine serum albumin (BSA) and incubated with primary antibodies O/N, anti-AQP2 (Pre-C-tail Ab) and anti-AQP2-pS256. Immunoreactive bands were detected with secondary goat anti-rabbit horseradish peroxidase-coupled antibodies obtained from Santa Cruz Biotechnologies (Tebu-Bio, Milan, Italy). Membranes were developed using Super Signal^®^ West Pico Chemiluminescent Substrate with the ChemiDoc System. Densitometry analysis was performed using Image Lab (Bio-Rad Laboratories, Milan, Italy). Data were summarized in histograms by using GraphPad Prism (GraphPad Software, San Diego, CA, USA).

### 4.7. Water Permeability Assay

Osmotic water permeability was measured by Video Imaging experiments. MCD4 cells were grown on 40 mm glass coverslips and loaded with 10 µM membrane permeable calcein green-AM for 45 min at 37 °C, 5% CO_2_ in DMEM. Cells were left under basal condition or stimulated with 100 nM dDAVP for 1h and/or treated with 100 nM lixivaptan for 1h. The coverslips with dye-loaded cells were mounted in a perfusion chamber (FCS2 Closed Chamber System, BIOPTECHS, Butler, PA, USA) and measurements were performed using an inverted microscope (Nikon Eclipse TE2000-S microscope), equipped for single cell fluorescence measurements and imaging analysis. The sample was illuminated through a 40× oil immersion objective (numerical aperture NA = 1.30). The calcein green-AM loaded sample was excited at 490 nm. Emitted fluorescence was passed through a dichroic mirror, filtered at 515 nm (Omega Optical, Brattleboro, VT, USA) and captured by a cooled ECCD camera (CoolSNAP HQ, Photometrics, Tucson, AZ, USA). Fluorescence measurements, following iso- (290 mOsm; 140 mM NaCl, 5 mM KCl, 1 mM MgCl_2_, 1 mM CaCl_2_, 10 mM Hepes, 5 mM Glucose) or hyperosmotic (460 mOsm; isosmotic solution added with 135 mM Mannitol) solutions, were carried out using Metafluor^®^ software v7.8.1.0 (Molecular Devices, LLC, San Jose, CA, USA). Calcein-AM is a nonfluorescent membrane-permeable dye, capable of quenching, which is converted to a green-fluorescent dye after acetoxymethyl ester hydrolysis, elicited by intracellular esterases. The exposure to a hyperosmotic solution leads to water efflux causing cell shrinkage with a consequent increase in calcein concentration, quenching, and ultimate decrease of fluorescence intensity. The best-fit tau values of the fluorescence intensity curve is proportional to the speed of water efflux and represents an indirect indication of the water permeability through AQP2 [16]. The time course of cell shrinkage was measured as time constant (*Ki*, s^−1^). 

### 4.8. Statistical Analysis

One-way ANOVA followed by Newman–Keuls multiple comparisons test was used for the statistical analysis. All values are expressed as means ± SEM. A difference of *p* < 0.05 was considered statistically significant.
